# Synergistic effects of herbal medicines and anticancer drugs: A protocol for systematic review and meta-analysis

**DOI:** 10.1097/MD.0000000000027918

**Published:** 2021-11-19

**Authors:** Chunhoo Cheon

**Affiliations:** Department of Preventive Medicine, College of Korean Medicine, Kyung Hee University, Seoul, Republic of Korea.

**Keywords:** anticancer drug, cancer, complementary and alternative medicine, herbal medicine, protocol, synergistic effect, systematic review

## Abstract

**Background:**

An increasing number of studies have been reporting combination therapy using herbal medicines and anticancer drugs, and the synergistic effects of this combination have gained much attention across the medical community. In this study, we will review and summarize all published studies that have investigated the synergistic interaction between herbal medicines and anticancer drugs.

**Methods:**

We will search the PubMed, Embase, and Cochrane Library databases. Studies investigated the synergistic interaction between herbal medicines and anticancer drugs will be included. The selection and extraction process will be performed by 2 independent reviewers, and we will perform qualitative synthesis.

**Discussion:**

The present study is being performed to investigate the herbal medicines and anticancer drugs that are used concomitantly, and to determine the combinations that are expected to show a synergistic effect. This knowledge will provide new insights into the possible role of herbal medicines in anticancer treatment.

**Review registration:**

Trial registration: OSF Registration number: DOI 10.17605/OSF.IO/H5QS9.

## 1 Introduction

Cancer is a leading cause of death worldwide, with an estimated 10 million deaths and a total estimated economic burden of 1.16 trillion US dollars in 2020.^[1]^ Significant research is ongoing globally in this field, and several studies are underway to determine the effects and mechanisms underlying the actions of herbal medicines used as anticancer drugs.^[2]^ Reportedly, the anticancer activity of herbal medicines is attributable to promotion of autophagic cell death, apoptosis, modulation of the tumor microenvironment, and suppression of tumor angiogenesis.^[3–6]^

Currently, many studies are being performed to investigate the effects of combination therapy using various anticancer drugs to overcome the limitations of known anticancer medications, and these drugs are also being administered to patients with cancer in real-world clinical practice.^[7]^ Although only a small percentage of studies have discussed this subject, co-administration of herbal medicines and anticancer drugs is being reported, and some of these combinations are shown to be effective.^[8]^

To date, cancer research in herbal medicines has focused on the following key issues: (a) the anticancer effect of herbal medicines and, (b) management of accompanying symptoms or the adverse effects of anticancer drugs in patients with cancer. Currently, no herbal medicine is approved as an anticancer drug, although several candidate agents are being investigated. The role of herbal medicines in the management of symptoms of cancer is primarily being explored in East Asia.^[9,10]^

Co-administration of herbal medicines and anticancer drugs has been considered over several decades and is primarily aimed at symptom alleviation or for management of the adverse effects of anticancer drugs. Such combination treatment is also expected to show synergistic effects as anticancer therapy. Few studies have reported the synergistic effects, if any, of co-administration of herbal medicines and anticancer drugs, and the mechanisms underlying this interaction are largely unclear. Therefore, we will perform a systematic review (SR) of studies that have investigated the synergistic effects of co-administration of herbal medicines and anticancer drugs to determine the combinations of herbal medicines and anticancer drugs that are effective in patients with cancer.

## 2 Materials and methods

This SR is presented in accordance with the Preferred Reporting Items for Systematic Review and Meta-analysis Protocols recommendations.^[11]^ Details of the protocol for this SR are registered on Open Science Foundation and can be accessed at https://osf.io/h5qs9. Any amendments to the review plan will be presented in the paper reporting the results.

### 2.1 Eligibility criteria

We will select eligible studies based on the following criteria:

#### 2.1.1 Types of reviews

Both clinical and experimental studies will be included to determine the synergistic effects of herbal medicines and anticancer drugs used as combination therapy. This SR will only include published, peer-reviewed, and full-text articles written in English.

#### 2.1.2 Types of participants

We will include patients with cancer who received herbal medicines and anticancer drugs. Experimental animals and cells treated with combination therapy comprising herbal medicines and anticancer drugs will also be included.

#### 2.1.3 Types of interventions

All combinations of herbal medicines and anticancer drugs will be investigated in this study. Articles that describe specific compounds extracted from herbal medicines will be excluded.

#### 2.1.4 Types of comparisons

There will be no restrictions with regard to the types of controls used in studies, provided that the studies report synergistic interactions between herbal medicine and anticancer drugs. Subgroup analysis will be performed for studies that discuss monotherapy versus combination therapy.

#### 2.1.5 Types of outcomes

The present study includes research that discusses the cytotoxicity or tumor response rate as outcome variables.

### 2.2 Search strategy

We intend to search the PubMed, Embase, and Cochrane Library databases for all relevant articles using the following free text keywords and Medical Subject Heading terms: “Antineoplastic Agents,” “Phytotherapy,” and “Drug Synergism.” Table 1 shows an example of the search strategy we intend to utilize. This example illustrates the strategy for the PubMed database; we will use a similar search strategy for the other aforementioned databases.

**Table 1:** Search keywords in PubMed.

Number	Search terms
1	Antineoplastic agents [MeSH Terms]
2	Anticancer agent
3	Anticancer drug
4	Chemotherapy
5	Antitumor drug
6	Antitumor agent
7	Phytotherapy [MeSH Terms]
8	Herbal medicine
9	Botanical drug
10	Natural product
11	Herbal therapy
12	Herb therapy
13	Drug synergism [MeSH Terms]
14	synergism
15	Drug augmentation
16	Drug Potentiation
17	Synergistic effect
18	Synergistic interaction

### 2.3 Review selection

**Figure 1:**
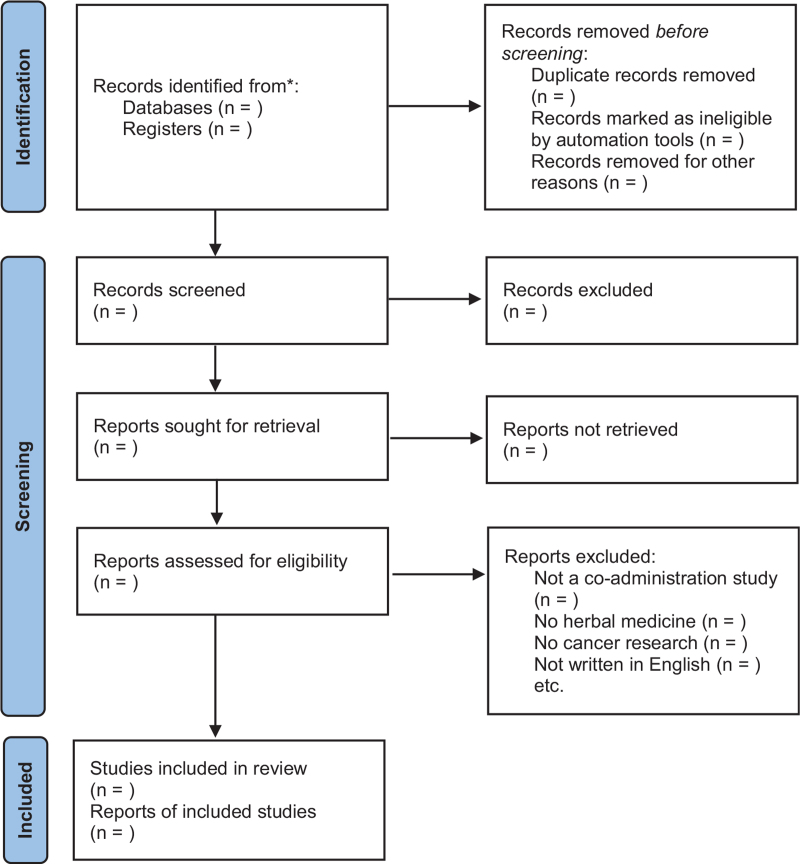
Flow diagram of study selection process.

Following a comprehensive search of all databases, we will carefully review the titles and authors to discard duplicate articles, and 2 independent reviewers will perform the final selection. Titles and abstracts of the selected articles will be screened, and studies that do not conform to the objectives of this SR will be excluded. Reviewers will carefully scrutinize the entire text and select the appropriate articles based on inclusion and exclusion criteria. Disagreements will be resolved through discussion to reach a consensus. Flow diagram of this study was presented in Figure 1.

### 2.4 Data extraction

The following information will be extracted from each SR: first author, year of publication, number of participants, participant characteristics, detailed descriptions of interventions performed, control groups, duration of interventions, outcome measures, and major findings. Data on experimental animals will be extracted in studies that describe animal models. We will provide the contents in the “comment” space for other detailed information. This process will be completed by 2 independent reviewers. We will contact the corresponding author via e-mail for information that is unavailable in the literature.

### 2.5 Data synthesis

In this study, we intend to initially perform qualitative analysis to target various carcinomas. Data of all included studies will be summarized in a tabular format. Continuous data will be presented as mean differences with 95% confidence intervals and categorical data as absolute and relative frequencies. Our study aims to investigate the herbal medicines that synergistically interact with anticancer drugs for the treatment of various cancers. The effects of each study will be synthesized in cases in which more than 2 clinical trials discuss the same carcinoma and drug. Data will be statistically analyzed using RevMan 5.4.1. Q statistics and *I*^2^ statistic will be used for measuring heterogeneity of included studies, when *I*^2^ < 50% and *P* > .10, the studies will be considered to have no substantial heterogeneity or less heterogeneity, and a fixed-effects model will be used, and vice versa, a random-effects model will be used. A revised Cochrane risk-of-bias tool for randomized trials will be used to evaluate the risk of bias in randomized trials.^[12]^ Funnel plot will be depicted to investigate publication bias.

### 2.6 Ethics issues

The present study is based on a review of previously published literature; therefore, Institutional Review Board approval is not required for the study.

## 3 Discussion

Despite significant advances in diagnostic and therapeutic approaches to cancer, this illness remains a challenge for humans, and several concerns and questions remain unresolved. Following the emergence of cytotoxic anticancer drugs (first-generation anticancer drugs), targeted anticancer drugs (second-generation anticancer drugs), and immuno-oncology drugs (third-generation anticancer drugs) studies are being widely performed to investigate the role of co-administration of various agents.^[13]^ Multidisciplinary cooperation is an essential component of technological progress and is applicable to the domains of traditional as well as Western medicine. Traditional medicine primarily includes herbal medicine, acupuncture, and moxibustion. Of these, acupuncture and moxibustion may slightly differ with regard to the acupuncture points and specific practice methods; however, they are relatively simple to classify and therefore, several studies have reported synthesis of evidence for these therapies.^[14–17]^ However, owing to the various compositions and effects of herbal medicines, compared to studies performed for other traditional medical therapies, it is difficult to perform evidence synthesis for herbal medicines and few evidence synthesis studies have specifically reported synergistic interactions of herbal medicines. Therefore, in the present study, we will review articles that discuss co-administration of herbal medicines and anticancer drugs, and studies that have reported the synergistic interactions between these agents.

Thus far, herbal medicines were administered to patients with cancer was to manage the accompanying symptoms and adverse effects of anticancer drugs. “Pattern differentiation,” a characteristic of traditional medicine, categorizes patients based on their symptoms, and the appropriate herbal medicines are prescribed depending on pattern differentiation. Symptom management is performed naturally following the application of pattern differentiation to patients with cancer.^[7]^ Although herbal medicines are useful for symptom management, additionally utilizing the anticancer properties of herbal medicines tends to maximize their potential. The anticancer actions of herbal medicines are increasingly being investigated, and several studies are being performed to confirm the effects of co-administration of herbal medicines and anticancer drugs.^[18,19]^ There is greater clarity regarding the mechanisms underlying the synergistic interactions between these agents, although further research is warranted to gain a deeper understanding of the subject.^[20–22]^ We expect that this study will generate interest regarding the anticancer properties of herbal medicines, particularly their synergistic interactions with anticancer drugs and will contribute to the development of novel herbal anticancer agents in future.

## Author contributions

**Conceptualization:** Chunhoo Cheon.

**Methodology:** Chunhoo Cheon.

**Writing – original draft:** Chunhoo Cheon.

**Writing – review & editing:** Chunhoo Cheon.

## References

[1] World Health Organization. Cancer, fact sheet. 2021; Available at: https://www.who.int/news-room/fact-sheets/detail/cancer. Accessed 19 October, 2021.

[2] Peng F Xie X Peng C. Chinese herbal medicine-based cancer therapy: novel anticancer agents targeting MicroRNAs to regulate tumor growth and metastasis. Am J Chin Med 2019;47:1711–35. 31801358 10.1142/S0192415X19500873

[3] Kim TW Cheon C Ko SG. SH003 activates autophagic cell death by activating ATF4 and inhibiting G9a under hypoxia in gastric cancer cells. Cell Death Dis 2020;11:717. 32879309 10.1038/s41419-020-02924-w PMC7468158

[4] Choi YJ Choi YK Lee KM Cho SG Kang SY Ko SG. SH003 induces apoptosis of DU145 prostate cancer cells by inhibiting ERK-involved pathway. BMC Complement Altern Med 2016;16:507. 27927199 10.1186/s12906-016-1490-5 PMC5142381

[5] Choi HS Kim MK Lee K. SH003 represses tumor angiogenesis by blocking VEGF binding to VEGFR2. Oncotarget 2016;7:32969–79. 27105528 10.18632/oncotarget.8808 PMC5078067

[6] Park CR Lee JS Son CG Lee NH. A survey of herbal medicines as tumor microenvironment-modulating agents. Phytother Res 2021;35:78–94. 32658314 10.1002/ptr.6784

[7] Bayat Mokhtari R Homayouni TS Baluch N. Combination therapy in combating cancer. Oncotarget 2017;8:38022–43. 28410237 10.18632/oncotarget.16723 PMC5514969

[8] Lee YK Bae K Yoo HS Cho SH. Benefit of adjuvant traditional herbal medicine with chemotherapy for resectable gastric cancer. Integr Cancer Ther 2018;17:619–27. 29614889 10.1177/1534735417753542 PMC6142080

[9] Cheon C Ko SG. A phase I study to evaluate the safety of the herbal medicine SH003 in patients with solid cancer. Integr Cancer Ther 2020;19:1534735420911442. 32186413 10.1177/1534735420911442 PMC7081467

[10] Chung VCH Wu X Lu P. Chinese herbal medicine for symptom management in cancer palliative care: systematic review and meta-analysis. Medicine (Baltimore) 2016;95:e2793. 26886628 10.1097/MD.0000000000002793 PMC4998628

[11] Moher D Shamseer L Clarke M. Preferred reporting items for systematic review and meta-analysis protocols (PRISMA-P) 2015 statement. Syst Rev 2015;4:01. 10.1186/2046-4053-4-1 PMC4320440 25554246

[12] Sterne JAC Savović J Page MJ. RoB 2: a revised tool for assessing risk of bias in randomised trials. BMJ 2019;366:l4898. 31462531 10.1136/bmj.l4898

[13] Carugo A Draetta GF. Academic discovery of anticancer drugs: historic and future perspectives. Ann Rev Cancer Biol 2019;3:385–408.

[14] Tan JB Wang T Kirshbaum MN. Acupoint stimulation for cancer-related fatigue: a quantitative synthesis of randomised controlled trials. Complement Ther Clin Pract 2021;45:101490. 34638053 10.1016/j.ctcp.2021.101490

[15] Liu W Lopez G Narayanan S. Acupuncture for cancer-related anorexia: a review of the current evidence. Curr Oncol Rep 2021;23:82. 33948746 10.1007/s11912-021-01067-1

[16] Guerra-Martín MD Tejedor-Bueno MS Correa-Casado M. Effectiveness of complementary therapies in cancer patients: a systematic review. Int J Environ Res Public Health 2021;18:03. 10.3390/ijerph18031017 PMC7908482 33498883

[17] Deng G Huang X Tu M. Efficacy and safety of moxibustion in the treatment of cancer-related fatigue: a protocol for systematic review and meta-analysis. Medicine (Baltimore) 2021;100:e24857. 33655946 10.1097/MD.0000000000024857 PMC7939204

[18] Cheon C Ko SG. Phase I study to evaluate the maximum tolerated dose of the combination of SH003 and docetaxel in patients with solid cancer: a study protocol. Medicine (Baltimore) 2020;99:e22228. 32957363 10.1097/MD.0000000000022228 PMC7505292

[19] Changou CA Shiah HS Chen LT. A Phase II clinical trial on the combination therapy of PHY906 plus capecitabine in hepatocellular carcinoma. Oncologist 2021;26:e367–73. 33140457 10.1002/onco.13582 PMC7930412

[20] Jeong MS Lee KW Choi YJ. Synergistic antitumor activity of SH003 and docetaxel via EGFR signaling inhibition in non-small cell lung cancer. Int J Mol Sci 2021;22:16. 10.3390/ijms22168405 PMC8395077 34445110

[21] Yang X Lam W Jiang Z. YIV-906 potentiated anti-PD1 action against hepatocellular carcinoma by enhancing adaptive and innate immunity in the tumor microenvironment. Sci Rep 2021;11:13482. 34188068 10.1038/s41598-021-91623-3 PMC8242098

[22] Liu S He X Man VH Ji B Liu J Wang J. New application of in silico methods in identifying mechanisms of action and key components of anti-cancer herbal formulation YIV-906 (PHY906). Phys Chem Chem Phys 2019;21:23501–13. 31617551 10.1039/c9cp03803e

